# Polymerization-Incompetent Uromodulin in the Pregnant Stroke-Prone Spontaneously Hypertensive Rat

**DOI:** 10.1161/HYPERTENSIONAHA.116.08826

**Published:** 2017-04-12

**Authors:** Sheon Mary, Heather Yvonne Small, Justyna Siwy, William Mullen, Ashok Giri, Christian Delles

**Affiliations:** From the BHF Glasgow Cardiovascular Research Centre, Institute of Cardiovascular and Medical Sciences, University of Glasgow, Scotland (S.M., H.Y.S., W.M., C.D.); Department of Biochemical Sciences, CSIR-National Chemical Laboratory, Pune, India (S.M., A.G.); and Mosaiques Diagnostics GmbH, Hannover, Germany (J.S.).

**Keywords:** hypertension, kidney, Nifedipine, pregnancy, uromodulin

## Abstract

The kidney is centrally involved in blood pressure regulation and undergoes extensive changes during pregnancy. Hypertension during pregnancy may result in an altered urinary peptidome that could be used to indicate new targets of therapeutic or diagnostic interest. The stroke-prone spontaneously hypertensive rat (SHRSP) is a model of maternal chronic hypertension. Capillary electrophoresis-mass spectrometry was conducted to interrogate the urinary peptidome in SHRSP and the control Wistar–Kyoto strain at three time points: prepregnancy and gestational days 12 and 18. The comparison within and between the Wistar–Kyoto and SHRSP peptidome at all time points detected 123 differentially expressed peptides (fold change >1.5; *P*<0.05). Sequencing of these peptides identified fragments of collagen α-chains, albumin, prothrombin, actin, serpin A3K, proepidermal growth factor, and uromodulin. Uromodulin peptides showed a pregnancy-specific alteration in SHRSP with a 7.8-fold (*P*<0.01) and 8.8-fold (*P*<0.05) increase at gestational days 12 and 18, respectively, relative to the Wistar–Kyoto. Further investigation revealed that these peptides belonged to the polymerization-inhibitory region of uromodulin. Two forms of uromodulin (polymerization competent and polymerization incompetent) were found in urine from both Wistar–Kyoto and SHRSP, where the polymerization-incompetent form was increased in a pregnancy-specific manner in SHRSP. Nifedipine-treated pregnant SHRSP showed only polymerization-competent uromodulin, indicating that calcium may be mechanistically involved in uromodulin polymerization. This study highlights, for the first time, a potential role of uromodulin and its polymerization in hypertensive pregnancy.

## Abstract 2

Supplemental Digital Content is available in the text.

Hypertensive complications are the most common clinical problems encountered during pregnancy.^1^ Hypertension during pregnancy encompasses many pathologies including preeclampsia, pregnancy-induced hypertension, and chronic hypertension. Specifically, chronic hypertension during pregnancy poses an increasing clinical problem.^2^ Pregnant women with chronic hypertension are at an increased risk of maternal and fetal morbidity and mortality, as well as a higher incidence of developing superimposed preeclampsia.^3^ The kidneys play a central role in blood pressure regulation in pregnancy. Women with chronic kidney disease are at increased risk of developing pregnancy complications, where up to 70% experience preterm delivery and up to 40% will develop preeclampsia.^4,5^ Significant structural and functional changes occur in the kidney during pregnancy including a 1- to 1.5-cm increase in size, a 50% increase in glomerular filtration rate, and up to an 80% increase in renal plasma flow.^6,7^ These alterations are broadly conserved in rats.^8^

The mechanisms that affect pregnancy-related changes in the kidney in normotensive and hypertensive women are incompletely understood. Unbiased screening approaches may have the ability to identify novel pathophysiological pathways. The urinary peptidome provides information about proteins that are involved in local processes in the kidneys and information about other organs obtained through filtration of the dynamic plasma peptidome.^9^ Peptides derived from processes in the kidney and urogenital tract form the majority of those detected in the urinary peptidome (70%), whereas peptides from the circulation constitute the remainder.^10^ The small peptides present are generally soluble and because of their size do not require protein digestion before analysis by mass spectrometry.^10^ Urinary peptidomics has been applied in the field of cardiovascular research to develop biomarker panels for diagnosis, prediction of disease, and risk stratification.^11^ In particular, many studies have shown that there are alterations in the urinary peptidome in people with hypertension^12^ and in healthy pregnant women compared with those who develop preeclampsia.^13^

The stroke-prone spontaneously hypertensive rat (SHRSP), obtained by selective inbreeding of the Wistar–Kyoto (WKY) strain, is a well-characterized model of human cardiovascular disease and of maternal chronic hypertension.^14^ We hypothesized that the urinary peptidome would be altered in both a pregnancy-dependent and strain-dependent manner between the SHRSP and control WKY strain.

## Methods

### Animals

Animals (WKY and SHRSP rats) were housed under controlled lighting (from 0700–1900 hours) and temperature (21±3°C) and received a normal diet (rat and mouse no. 1 maintenance diet; Special Diet Services, Grangemouth, United Kingdom) provided ad libitum. All animal procedures were approved by the Home Office according to regulations regarding experiments with animals in the United Kingdom (Project License Number 60/4286). Females were time mated at 12 weeks of age (±4 days). Nonpregnant animals were age matched at 15 weeks±4 days (ie, 12 weeks of age plus 21 days of pregnancy). Day 0 of pregnancy was defined as the day that a coital plug was observed indicative of successful mating having taken place. A subset of SHRSP began nifedipine treatment at 7 weeks of age at 25 mg/kg per day administered in 2 doses: a 10-mg/kg per day dose mixed in a 1-mL aliquot of baby food and a 15-mg/kg per day dose in drinking water to maintain lowered blood pressure throughout the 24-hour period. Stock solutions of nifedipine in drinking water were prepared in ethanol and diluted to the appropriate concentration with no more than a 0.8% final ethanol concentration. The number of rats and particular gestational day (GD) is given in the relevant figure legend.

### Metabolic Cage

The metabolic cage allows individual housing of an animal to collect information on water intake and urine output over 24 hours. A fixed amount of water (200 mL) was given, and food was available ad libitum over the 24-hour period. Animals were acclimatized for 4 hours, 3 days before measurement. For untreated WKY and SHRSP, urine samples were collected from virgin animals that were housed in the metabolic cage 1 day before mating and then at GD12 and GD18. For nifedipine-treated SHRSP, urine samples were collected at GD12 and GD18. Urine samples were aliquoted on the ice and stored at −80°C until use.

### Urinary Peptidomics

Seven hundred microliters of urine was diluted with 700 µL of 2 M urea and 0.1 M NH_4_OH containing 0.02% SDS. A size cutoff for peptides <20 kDa was performed using Centrisart ultracentrifugation filter devices (Sartorius, Göttingen, Germany) at 3000*g* for 1 hour at 4°C. To remove urea, electrolytes, and salts, the filtrate was then ran through a PD-10 desalting column (Amersham Bioscience, Buckinghamshire, United Kingdom) and peptide elution was done using 0.01% aqueous NH_4_OH. Finally, all samples were lyophilized, stored at 4°C, and resuspended in high-performance liquid chromatography grade H_2_O to a final concentration of 2 µg/µL before analysis.

CE-TOF-MS (capillary electrophoresis–time of flight–mass spectrometry) analysis was performed using a P/ACE MDQ CE system (Beckman Coulter, Fullerton,) online coupled to a micro-TOF MS (Bruker Daltonic, Bremen, Germany) as described in the study by Albalat et al.^15^ Samples were injected with 2 psi for 99 seconds (250 nL), and separation of peptides in the cartridge maintained at 25°C was attained at 25 kV for 30 minutes and increasing pressure (0.5 psi) for another 35 minutes. The sheath liquid consisted of 30% isopropanol, 0.4% formic acid in high-performance liquid chromatography grade water, and running buffer consisted of 79:20:1 (v/v) water, acetonitrile, and formic acid. The electrospray ionization sprayer (Agilent Technologies, CA) was grounded, and the ion spray inference potential was set at −4.5 kV. Spectra were accumulated over a mass:charge ratio of 350:3000 for every 3 seconds.

Peak picking, deconvolution, and deisotoping of mass spectral ion peaks were processed using Mosaiques Visu software.^16^ The CE-migration time, molecular weight, and ion signal intensity were normalized based on the reference signal from internal peptide standards/calibrants (peptides from housekeeping proteins) in rats.^17^ For calibration, a local and linear regression algorithm was applied with calibrants. The peak list generated for each peptide consisted of molecular weight (kDa), normalized CE migration time (minutes), and normalized signal intensity. The peptide list from all the samples that passed the quality control criteria was compared and annotated in a Microsoft SQL database. The criteria for clustering peptides in different samples were as follows: (1) molecular weight deviation less than ±50 ppm for small peptide (<800 Da) and gradually increasing to ±75 ppm for larger peptides (20 kDa), (2) CE-migration time deviation with linear increase from ±0.4 to ±2.5 minutes in the range from 19 to 50 minutes. Each peptide was given a unique identification number (Peptide ID). Peptides detected with the frequency of ≥70% in at least 1 group were considered for further analysis.

### Liquid Chromatography–Tandem Mass Spectrometry for Peptide Sequencing

The peptide mixtures extracted for CE-MS were also used for sequencing of the peptides in liquid chromatography–tandem mass spectrometry and CE-MS/MS. liquid chromatography–tandem mass spectrometry sequencing was performed on an UltiMate 3000 nanoflow system (Dionex/LC Packings) connected to a linear trap quadrupole Orbitrap hybrid mass spectrometer (Thermo Fisher Scientific, Germany) equipped with a nanoelectrospray ion source. After loading (5 µL) onto a Dionex 0.1×20 mm 5-µm C18 nanotrap column at a flowrate of 5 µL/min in 98% of 0.1% formic acid and 2% acetonitrile, sample was eluted onto an Acclaim PepMap C18 nanocolumn 75 µm × 50 cm, 2 µm 100 Å at a flow rate of 0.3 µL/min. The trap and nanoflow column were maintained at 35°C. The samples were eluted with a gradient of solvent A: 98% water, 0.1% formic acid, and 2% acetonitrile versus solvent B: 80% acetonitrile, 20% water, 0.1% formic acid starting at 1% B for 5 minutes rising to 20% B after 90 minutes and finally to 40% B after 120 minutes. The column was then washed and re-equilibrated before the next injection. Alternatively, samples were injected and separated using a P/ACE MDQ capillary electrophoresis system (Beckman Coulter, Fullerton) as described above for CE-MS.

The eluent from the LC or CE was ionized using a Proxeon nanospray electrospray ionization source operating in positive ion mode into an Orbitrap Velos FTMS (Thermo Finnigan, Bremen, Germany). Ionization voltage was 2.6 kV, and the capillary temperature was 250°C. The mass spectrometer was operated in MS/MS mode scanning from 380 to 1600 amu. In LC, the top 20 multiply charged ions were selected from each scan for MS/MS analysis using HCD at 40% collision energy. The resolution of ions in MS1 was 60 000 and 7500 for HCD MS2. In CE, the top 5 multiply charged ions were selected for MS/MS using a data-dependent decision tree method^18^ and fragmented by either HCD at 40% or electron-transfer dissociation, depending on their mass and charge state.

MS and MS/MS data files were searched, in this case, against the Uniprot rat nonredundant database using SEQUEST (Thermo Proteome Discoverer) with the nonspecific enzyme as enzyme specificity. Peptide data were extracted using high peptide confidence and top 1 peptide rank filters. A peptide mass tolerance of ±10 ppm and a fragment mass tolerance of ±0.05 Da.

### Quantitative Polymerase Chain Reaction for Uromodulin Expression

Gene expression assay for uromodulin (*Umod*) in kidney tissues was performed using the following probes from Thermo Fisher, Paisley, United Kingdom: *Umod* (Rn01507237_m1) and *Actb* (4352340E). Ct values were analyzed using the 2^(-ΔΔCt)^ method, with ΔCt indicating normalization to the housekeeper β-actin (*Actb*).

### Western Blot for Umod

Multistrip blotting was performed as described previously.^19^ Primary and secondary antibodies were used as per manufacturer’s instructions. Primary antibody Umod (AF5175; R&D Systems, Abingdon, United Kingdom) followed by secondary antibody anti-sheep horseradish peroxidase conjugate (HAF016; R&D Systems).

### N-Deglycosylation of Umod

Umod was N-deglycosylated by PNGase F (New England Biolabs, Beverly, MA) under reducing conditions. 2 µg of Umod was denatured with buffer provided by the manufacture and then incubated with PNGase F at 37°C for 1 hour. Protein samples were separated on reducing 4% to 12% NuPAGE gel and later blotted onto polyvinylidene fluoride membrane for Umod detection.

### Purification of Umod and Polymerization Assay

Urine samples from untreated WKY (n=7), SHRSP (n=7), and nifedipine-treated SHRSP (n=3) at prepregnancy, GD12, and GD18 were pooled separately. For purification, 500 µL of urine was filtered using a 3000 Da molecular weight cutoff column (Millipore). Polymerization assay was performed as described previously by Jovine et al.^20^ Pellet and supernatant were solubilized in SDS-gel loading buffer and separated on a reducing 10% NuPAGE gel and blotted onto polyvinylidene fluoride membrane for Umod detection.

### Protease Prediction

To find the protease that cleaved Umod protein in vivo, Proteasix software was used to perform in silico protease mapping as described in the study by Klein et al.^21^

### Statistical Analysis

In urinary peptidomics, peptides were considered significant according to Wilcoxon rank-sum test (*P*<0.05) followed by adjustment for multiple testing (Benjamini and Hochberg). Later repeated-measure ANOVA was used to evaluate significant peptides within different GDs. Western blot analysis was performed in LI-COR Image Studio software, and band intensities were made consistent with local background subtraction. Furthermore, *t* test, 1-way, or 2-way ANOVA test were used for statistical significance (*P*<0.05).

## Results

### Urinary Peptidome Is Altered During Pregnancy and Between WKY and SHRSP

**Figure 1.**
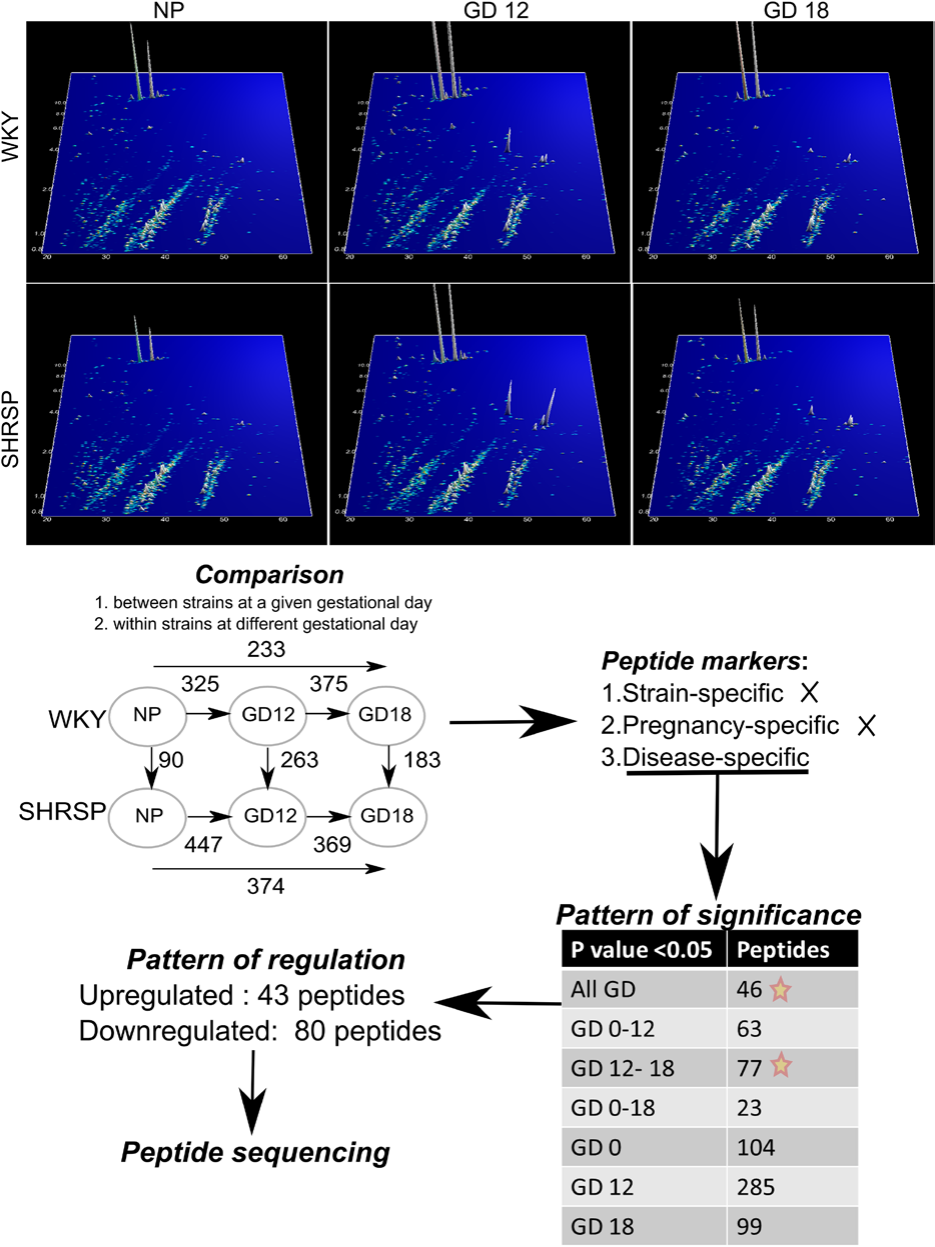
Schematic flowchart representing peptidomics data analysis and identification of peptide markers. The counterplot at the **top** represents the peptide mass fingerprint pattern of Wistar–Kyoto (WKY; n=7) and stroke-prone spontaneously hypertensive rat (SHRSP); n=7) at prepregnancy (nonpregnant [NP]) and gestational days (GDs) 12 and 18 observed in capillary electrophoresis–mass spectrometry. On the plot, *X*-axis represents the CE-migration time, *Y*-axis mass:charge ratio, and *Z*-axis the peptide signal intensity. Various comparisons were made between strains and GDs. The numbers above the arrow represent the number of peptide identified during the comparison with *P*<0.05. The strain- and pregnancy-specific peptide markers were not used to for analysis. The disease-specific peptide markers were analyzed for pattern of significance, that is, at *P*<0.05 whether the peptide was significant at a given GD. Star marked pattern of significance represents peptides that were significant at all GD or GDs 12–18 and were further used for analysis.

A peptidome screen was performed in WKY and SHRSP urine at prepregnancy, GD12, and GD18 to identify strain, pregnancy, and disease-dependent alterations (Figure 1). The peptidomic data were subject to several comparisons between WKY and SHRSP at different GDs, such as (1) comparison within rat models at all time points and (2) comparison between WKY and SHRSP at a given GD. The longitudinal comparison (1) within the WKY and SHRSP resulted in the identification of 630 and 739 significant differentially regulated peptides, respectively. These were considered to be strain- and pregnancy-dependent alterations. Although the comparison between WKY and SHRSP (2) resulted in 788 significant peptides that were considered to be hypertension-dependent alterations. These disease-specific peptide markers were further evaluated using repeated-measures ANOVA. Some peptides were significantly altered at all time points, or at any two or at a single GD. The peptides that showed significance at all time points and at both GD12 and GD18 were considered for further analysis. These 123 peptides were investigated for their regulation pattern with cutoff criteria of ≥1.5-fold change and *P*≤0.05 (Table S1 in the online-only Data Supplement). Compared with WKY, urine from SHRSP consisted of 7 and 39 peptides up- and downregulated, respectively, at prepregnancy, GD12, and GD18. In addition, 36 peptides were upregulated and 41 peptides were downregulated in SHRSP at both GD12 and GD18. Sequencing of these differentially expressed peptides revealed that they belonged to collagen α-chains, albumin, prothrombin, actin, serpin A3K, proepidermal growth factor, and Umod (Table S2).

### Umod Expression Is Increased in the Urine and Kidney of SHRSP During Pregnancy

**Figure 2.**
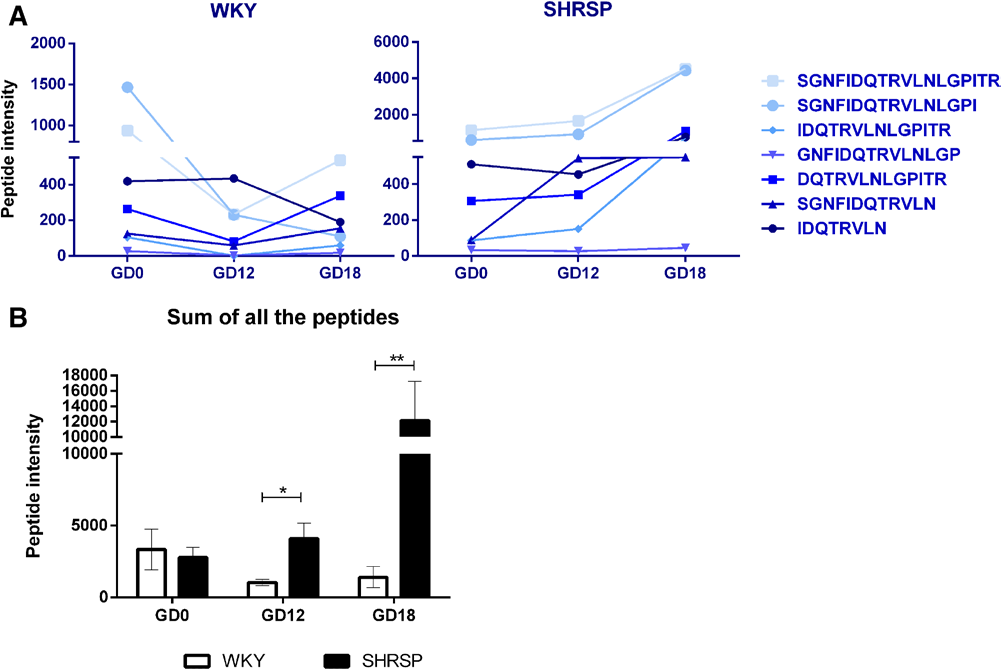
Urinary Umod peptides are increased in the stroke-prone spontaneously hypertensive rat (SHRSP) relative to Wistar–Kyoto (WKY) in a pregnancy-dependent manner. Seven peptides detected in the urinary peptidome were derived from Umod protein (**A**). Of these peptides, they were all increased in a pregnancy-specific manner in SHRSP (n=7) relative to WKY (n=7) at gestational days (GDs) 12, GD18, or GD12 and GD18. Taking into account the sum of all of the 7 peptides (**B**) showed that Umod peptides were increased in a pregnancy-specific manner at GD12 and GD18 in SHRSP relative to WKY (*P<0.05, **P<0.01 vs WKY analyzed by Wilcoxon rank test).

**Figure 3.**
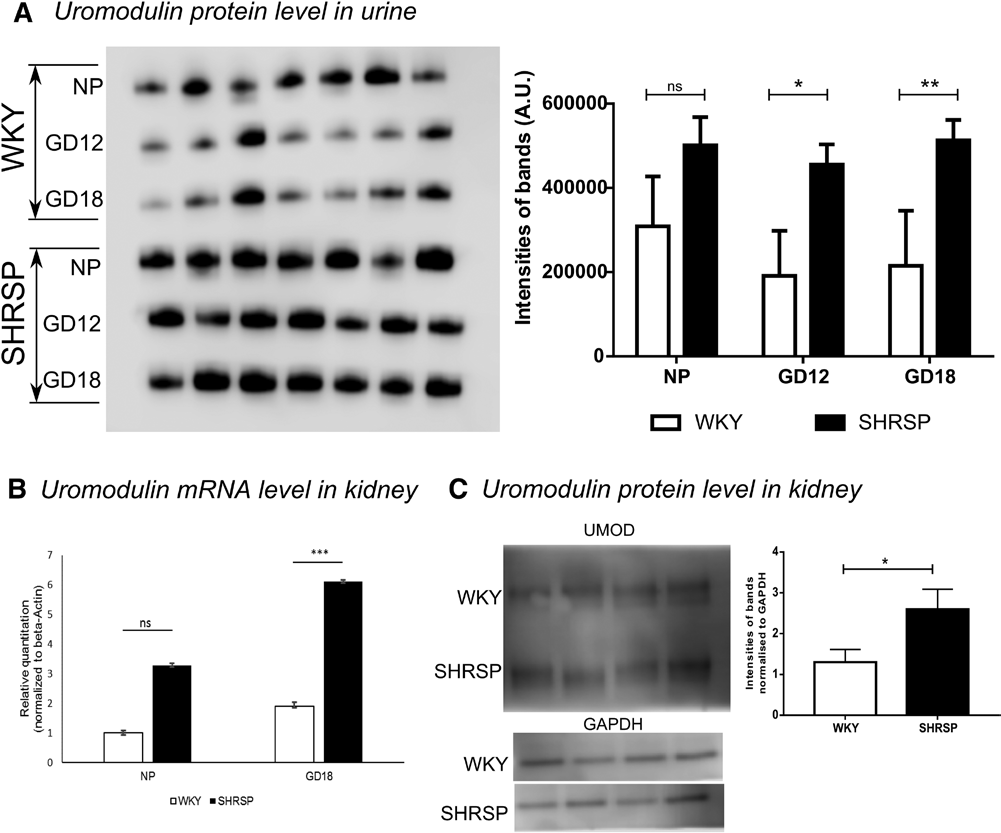
Increase in Umod in stroke-prone spontaneously hypertensive rat (SHRSP) validated in urine and kidney tissue. **A**, Purified Umod from the urine of Wistar–Kyoto (WKY; n=7) and SHRSP (n=7) was run on 10% NuPAGE gel and blotted onto a single polyvinylidene fluoride membrane. Umod showed an increase in SHRSP in pregnancy-dependent manner at gestational days (GDs) 12 and 18. **B**, Gene expression of *Umod* was measured in kidney tissue from nonpregnant and pregnant (GD18) WKY and SHRSP (n=5). *Umod* expression was increased in kidney tissue from SHRSP at both NP (*P*=n.s.) and GD18 time points (*P*<0.001). **C**, Umod protein was measured from kidney tissue extract of pregnant (GD18) SHRSP (n=4) and WKY (n=4). Pregnant SHRSP showed increased Umod expression in kidney tissue (**P<0.01, ***P<0.001 vs WKY analyzed by 1-way ANOVA, 2-way ANOVA, and *t* test).

The CE-MS data indicated that Umod peptide expression was greater in SHRSP urine samples relative to WKY with a fold change of 4 (*P*<0.05) and 8 (*P*<0.01) at GD12 and GD18, respectively (Figure 2A and B). These data were validated in individual urine samples from WKY and SHRSP at prepregnancy, GD12, and GD18 with Western blot where urine from SHRSP showed an increase in Umod protein expression at GD12 and GD18 with a fold change of 2.3 (*P*<0.05; Figure 3A).

Gene expression of *Umod* was measured in kidney tissue taken from nonpregnant and GD18 WKY and SHRSP. *Umod* gene expression was greater in kidney tissue from SHRSP both in nonpregnant and GD18 samples, but the difference only reached statistical significance at GD18 (*P*<0.001; Figure 3B). This finding was validated at the protein level, which showed greater levels of Umod in kidney tissue from GD18 SHRSP (Figure 3C).

### C-Terminal Umod Peptides Found to Be More Abundant at GD12 and GD18 in SHRSP Urine

**Figure 4.**
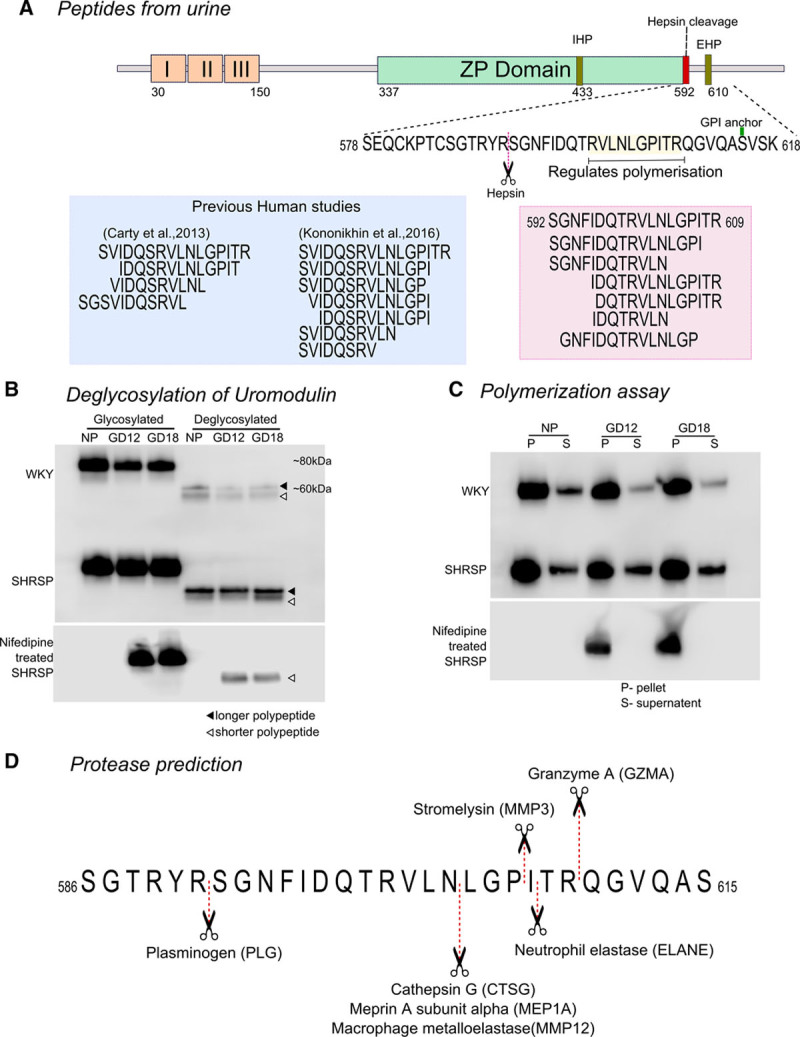
**A**, Schematic representation of rat Umod structure containing an epidermal growth factor–like domain (orange box I, II, and III), the Zona Pellucida (ZP) domain, internal and external hydrophobic patches (IHP and EHP, respectively), hepsin cleavage site and glycosylphosphatidylinositol (GPI) anchoring site. The zoomed-in sequence represents the C-terminal region identified in mass spectrometry rat data (pink) and previous human studies on preeclampsia (blue). **B**, Deglycosylation of Umod identified 2 bands in untreated WKY (pool of n=7) and stroke-prone spontaneously hypertensive rat (SHRSP; pool of n=7) at all gestational day (GD), and only single band in nifedipine-treated SHRSP (pool of 3) at GD12 and GD18. **C**, In the polymerization assay, the pellet fraction (P) represents the polymerization-competent and supernatant (S) the polymerization-incompetent Umod. Polymerization assay showed polymerization-incompetent Umod in the supernatant (S) of untreated WKY (pool of n=7) and SHRSP (pool of n=7) at all GDs, whereas no Umod bands were observed in nifedipine-treated SHRSP (pool of 3). Polymerization-competent Umod in the pellet (P) was observed in untreated WKY and SHRSP, as well as in nifedipine-treated SHRSP. **D**, Proteasix software predicted few serine proteases and metalloproteases that might cleave the C-terminal of Umod, which resulted in the peptides observed in urine. CTSG indicates cathepsin G; ELANE, neutrophil elastase; GZMA, granzyme A; MEP, Meprin A subunit α; MMP, matrix metalloprotease; NP, nonpregnant; and PLG, plasminogen.

CE-MS analysis identified 7 peptides of Umod present in the urine samples from WKY and SHRSP. Further sequencing of these peptides using liquid chromatography-tandem mass spectrometry revealed that they were exclusively derived from the same C-terminal region (592–609; Figure 4A). This region of Umod remains membrane bound after the formation of extracellular polymerization-competent Umod and is also known to have an inhibitory role in Umod polymerization. Expression of these peptides was significantly greater at GD12, GD18, or both in SHRSP relative to WKY. When compared with our group’s previous study that examined urinary peptidomics in women with preeclampsia, we found the ortholog peptides of Umod upregulated in preeclampsia.^22^ In another independent study by Kononikhin et al^23^ that examined the urinary peptidome in mild and severe preeclampsia, the same sequence of peptides of Umod was identified as early predictors of preeclampsia (Figure 4A). The presence of these peptides in urine suggests that either they are cleaved by certain proteases from the membrane after the formation of extracellular polymerization-competent Umod or from a longer form of Umod that retains this region. We hypothesized that 2 forms of Umod exist in the urine of the pregnant rat: a shorter polymerization-competent and a longer polymerization-incompetent form.

### Polymerization-Incompetent Umod Is Increased in Pregnant SHRSP

N-deglycosylation of Umod using PNGase F confirmed the presence of 2 forms of Umod: a longer polypeptide (≈59 kDa) and a shorter polypeptide (≈54 kDa; Figure 4B). The presence of the 59-kDa polypeptide of Umod was increased in the urine of SHRSP compared with WKY at all time points (Figure 4B). The polymerization assay identified Umod both in the supernatant (polymerization incompetent) and pellet (polymerization competent) in both strains (Figure 4C). In nonpregnant rats, both polymerization-competent and polymerization-incompetent Umod were detected in WKY and SHRSP at similar levels (Figure 4C). On pregnancy, the polymerization-incompetent Umod decreased in a gestation-dependent manner in the WKY (Figure 4C). In contrast, the polymerization-incompetent form of Umod increased over pregnancy in the SHRSP (Figure 4B and 4C).

### Nifedipine-Treated Pregnant SHRSP Showed Only Polymerization-Competent Umod

To determine whether the presence of hypertension altered the peptidomic profile of Umod in the SHRSP, urine samples were analyzed from SHRSP treated with nifedipine from 7 weeks of age (Figure S1). Urine samples from pregnant nifedipine-treated SHRSP showed that nifedipine-treated rats had only a single band of N-deglycosylated Umod in Western blot (Figure 4B). In the polymerization assay from these samples, Umod was present only as the polymerization-competent form, whereas the polymerization-incompetent form was undetectable at both GD12 and GD18 (Figure 4C).

### Protease Activity on Polymerization-Incompetent Umod

When analyzing peptides of Umod, it is important to understand how these were derived from the full-length protein through the action of various proteases. Proteasix software^21^ was used to predict the proteases that might be responsible for cleaving the polymerization-incompetent Umod at the C-terminal in silico. Most of these predicted proteases were classified as either a serine protease or a metalloprotease. Meprin A subunit α, a metallopeptidase was predicted with medium confidence, whereas other proteases such as granzyme A, cathepsin G, matrix metalloproteases 3 and 12, plasminogen, and neutrophil elastase were predicted with lower confidence (Figure 4D; Table S3).

## Discussion

Interrogation of the urinary peptidome over gestation in WKY and SHRSP showed that there are strain-dependent and pregnancy-dependent alterations. To identify relevant peptides, we focused on 123 peptides that were found to be differentially expressed between WKY and SHRSP at all time points (nonpregnant, GD12, and GD18) or at GD12 and GD18 only. These 123 peptides were principally composed of collagen α chains, serum albumin, prothrombin, actin, serpin A3K, proepidermal growth factor, and Umod. In comparison, in urinary peptidomic screens of women with preeclampsia, the most common constituents are albumin and tubular proteins that are thought to reflect renal tubule damage.^24^ However, a characteristic and specific signature for human preeclampsia are yet to be determined despite many studies.^24^ The nonbiased peptidome screening of urine collected prepregnancy, GD12 and GD18 led to the identification of Umod peptides that were increased in a pregnancy-dependent manner in SHRSP relative to WKY. Further investigation of these peptides revealed that they were all derived from the polymerization-inhibitory region of Umod. In keeping with this finding, Umod polymerization was altered between WKY and SHRSP, specifically the polymerization-incompetent form of Umod was increased in pregnant SHRSP. Our data are the first to introduce a role of polymerization of Umod in hypertensive pregnancy.

Umod has been extensively studied in association with cardiovascular conditions in humans. Genome-wide association studies have identified *UMOD* variants associated with renal function and hypertension.^25^ However, the role of Umod in hypertensive pregnancy has not yet been subject to detailed study. All 7 of the urinary Umod peptides detected in the present screen were increased in a pregnancy-dependent manner in the SHRSP relative to the WKY. The SHRSP pregnancy-dependent increase in Umod was validated by increased *Umod* gene and protein expression in kidney tissue at GD18. Umod protein expression in urine of WKY rats showed decrease over pregnancy (nonsignificant), whereas in SHRSP, its expression is increased significantly. One limitation of our model is that urinary Umod increases with gestation in human pregnancy, whereas there is a decrease in urinary Umod in the WKY. Umod is the most abundant protein in the urine, secreted by the epithelial cells lining the thick ascending limb of the loop of Henle in the kidney. The full-length Umod in endoplasmic reticulum gets N-glycosylated and glypiated at its C terminus and further modified in Golgi apparatus. The mature Umod with glycosylphosphatidylinositol modification (at S615) is anchored to the apical membrane of thick ascending limb facing the tubular lumen. The secreted form of Umod is released by the proteolytic activity of hepsin, a type II transmembrane serine protease (at R591 in rat evidenced by sequence similarity).^26^ Polymerization of secreted Umod helps in the formation of a filamentous gel-like structure that acts a physical barrier for ion transport to maintain countercurrent gradients in the interstitium.^27,28^ Cleavage by hepsin releases the polymerization-inhibitory motif (extracellular hydrophobic patch) that prevents premature intracellular protein assembly.^26,29^

Urinary peptidomics presented in this article indicated that the Umod peptide 592-SGNFIDQTRVLNLGPITR-609 and its smaller fragments were released into the urine. We found these peptides upregulated in pregnant SHRSP rats, as well as in urine samples from 2 independent cohorts of women with preeclampsia in other work.^22,23^ This sequence 592 to 609 consisted of the polymerization-inhibitory motif (601–610), downstream of the hepsin cleavage site (R591) and upstream of glycosylphosphatidylinositol anchoring. This led to the question as to whether these peptide fragments were released from the membrane after hepsin cleavage or were these fragments cleaved from a longer form of Umod polypeptide. To address this, N-deglycosylation of Umod was performed where 2 polypeptides were identified. The polymerization assay confirmed the existence of 2 forms of Umod, the longer polymerization-incompetent and shorter polymerization-competent polypeptides.

The longer polymerization-incompetent Umod was observed in nonpregnant WKY and SHRSP. This indicates that the release of a longer polypeptide is a common phenomenon in these strains. However, during pregnancy, the release of the longer polypeptide is higher in SHRSP than in WKY. This indicates that there is less Umod polymerization in SHRSP. We hypothesize that the longer polypeptides are later cleaved by other as yet unidentified proteases to form the shorter polypeptide, which releases the peptides that were observed in the urinary peptidome. Furthermore, the activity of the predicted proteases should be increased in SHRSP during pregnancy corresponding to the higher levels of the 7 protein fragments of Umod observed in the urinary peptidome data. The preliminary prediction data generated using Proteasix identified many candidate proteases for further study.

The correlation between hypertension during pregnancy and release of Umod peptides warrants further investigation. We made an attempt to explore the effect of blood pressure on Umod polymerization when pregnant SHRSP were treated with the antihypertensive drug, nifedipine, from 7 weeks of age. It was found that nifedipine-treated rats expressed only the polymerization-competent Umod. Nifedipine principally acts as a calcium channel blocker and is known to reduce urinary protein excretion rate in patients with renal disease^30^ and decrease urinary calcium excretion in women with preeclampsia.^29^ In this study, nifedipine treatment significantly lowered the blood pressure of the pregnant SHRSP. The effect of nifedipine indicates 2 possible mechanisms that drive Umod polymerization. First, the role of calcium in regulating the polymer formation, and second, the existence of an indirect unknown mechanism that modulates hypertension and polymer formation. Umod is known to have 3 epidermal growth factor–like domains at the N terminus where 2 of these domains, epidermal growth factor–like 2 (D67-I108) and epidermal growth factor–like 3 (D109-E150), are calcium-binding domains that have implications in protein–protein interaction. Umod is also known to play a protective role against calcium crystal formation.^31^ The predicted proteases such as granzyme A, matrix metalloproteinase matrix metalloproteases 3 and 12, metalloendopeptidase, neutrophil elastase, plasmin are all dependent on calcium for their activity, expression, or structure.^32–36^ These proteases were derived from in silico analysis in the current study. Future work on determining the expression of each of these proteases, and their role in Umod cleavage should be undertaken in future studies.

## Perspectives

SHRSP exhibit strain-dependent and pregnancy-dependent alterations in their urinary peptidome relative to the WKY. The protein Umod, which has already been shown to have an important role in systemic hypertension, has been highlighted in this study as a potential protein of interest in hypertensive pregnancy, especially in terms of its polymer formation function. At this stage, it is not clear whether Umod polymerization is a counter-regulatory mechanism or secondary to the development of hypertension. These findings warrant future work to reciprocate these findings in human samples and the molecular dissection of Umod polymerization during healthy and hypertensive pregnancy.

## Acknowledgments

We would like to thank Dr Markus P. Schneider for critically reviewing this article.

## Sources of Funding

This study was funded by grants from the Commonwealth Scholarship Commission (reference INCN-2015–20) to S. Mary, a British Heart Foundation Student Fellowship (FS/12/66/30003) to H.Y. Small, the European Union (sysVASC; project reference 603288) and a British Heart Foundation Centre of Research Excellence Award to C. Delles.

## Disclosures

J. Siwy is employee of Mosaiques Diagnostics GmbH. The other authors report no conflicts.

## Supplementary Material

## Novelty and Significance

### What Is New?

- This is the first study to identify a role for uromodulin polymerization in hypertensive pregnancy.
- We identified elevated levels of uromodulin peptides in the urine of pregnant stroke-prone spontaneously hypertensive rat relative to the control Wistar–Kyoto rat.
- The pregnant stroke-prone spontaneously hypertensive rat shows elevated levels of polymerization-competent uromodulin, which is significantly reduced with antihypertensive treatment.

### What Is Relevant?

- Hypertensive pregnancy is the most common pregnancy complication encountered and contributes substantially to maternal and fetal morbidity and mortality.
- Uromodulin is extensively studied in relation to systemic arterial hypertension; here, we report its role in hypertensive pregnancy.

### Summary

Pregnant stroke-prone spontaneously hypertensive rat exhibit an altered urinary peptidome relative to Wistar–Kyoto rats. Uromodulin is associated with hypertension and other cardiovascular disorders in humans and preclinical models. Elevated levels of polymerization-incompetent uromodulin were seen in pregnant stroke-prone spontaneously hypertensive rat, which were not detectable in nifedipine-treated pregnant stroke-prone spontaneously hypertensive rat. The role and mechanism of polymerization of uromodulin in hypertension and hypertensive pregnancy warrants further study in human subjects.
