# Supplementary material for: Polymerization-Incompetent Uromodulin in the Pregnant Stroke-Prone Spontaneously Hypertensive Rat
Source: Hypertension. 2017 Apr 12;69(5):910–8. doi: 10.1161/HYPERTENSIONAHA.116.08826 (PMC5389592; doi:10.1161/HYPERTENSIONAHA.116.08826)
Supplement: Supplementary file 1 [file hyp-69-910-s001.docx]

**SUPPLEMENTAL MATERIAL**

**Polymerization-incompetent Uromodulin in the Pregnant Stroke Prone Spontaneously Hypertensive Rat**

**Sheon Mary^1, 2^ *, Heather Yvonne Small^1^ *, Justyna Siwy^3^, William Mullen^1^, Ashok Giri^2^, Christian Delles^1^**

^1^BHF Glasgow Cardiovascular Research Centre, Institute of Cardiovascular and Medical Sciences, University of Glasgow, Scotland

^2^Department of Biochemical Sciences, CSIR-National Chemical Laboratory, India

^3^Mosaiques Diagnostics GmbH, Hannover, Germany

* SM and HYS contributed equally to this work.

**Corresponding author:** Prof. Christian Delles

Address: BHF Glasgow Cardiovascular Research Centre, Institute of Cardiovascular and Medical Sciences, University of Glasgow, 126 University Place, Glasgow, G12 8TA

Telephone number: 01413302749

Email: Christian.Delles@glasgow.ac.uk

# Table S1: 123 urinary peptides altered between WKY and SHRSP at all time points (NP, GD12 and GD18) or GD12 & GD18 only

| **PeptideID** | **Mass [Da]** | **Migration time [Min]** | **Log2 peptide intensity** | | | | | | **Fold change (WKY/ SHRSP)** | | |
| --- | --- | --- | --- | --- | --- | --- | --- | --- | --- | --- | --- |
|  |  |  | **WKY**  **NP** | **SHRSP**  **NP** | **WKY**  **GD12** | **SHRSP**  **GD12** | **WKY**  **GD18** | **SHRSP**  **GD18** | **NP** | **GD12** | **GD18** |
| 929 | 857.4815 | 28.15 | 5.93 | 6.28 | 6.35 | 7.03 | 5.97 | 7.12 | 0.94 | 0.90 | 0.84 |
| 1054 | 862.4315 | 22.67 | 6.57 | 8.10 | 8.11 | 6.86 | 6.86 | 6.36 | 0.81 | 1.18 | 1.08 |
| 1304 | 874.4576 | 34.49 | 7.49 | 6.14 | 7.73 | 5.75 | 6.64 | 3.95 | 1.22 | 1.34 | 1.68 |
| 4119 | 1009.498 | 37.80 | 12.33 | 13.33 | 12.23 | 12.91 | 11.82 | 13.09 | 0.92 | 0.95 | 0.90 |
| 4706 | 1046.479 | 47.56 | 5.75 | 6.67 | 5.57 | 7.32 | 6.13 | 4.75 | 0.86 | 0.76 | 1.29 |
| 5098 | 1073.363 | 46.52 | 13.04 | 13.67 | 13.11 | 13.83 | 13.28 | 14.25 | 0.95 | 0.95 | 0.93 |
| 5254 | 1083.533 | 36.50 | 11.96 | 11.35 | 12.24 | 11.21 | 12.08 | 11.42 | 1.05 | 1.09 | 1.06 |
| 5474 | 1099.53 | 36.52 | 8.98 | 7.83 | 9.31 | 7.72 | 9.86 | 8.23 | 1.15 | 1.21 | 1.20 |
| 5497 | 1100.587 | 28.83 | 7.31 | 5.11 | 8.58 | 7.67 | 8.52 | 6.09 | 1.43 | 1.12 | 1.40 |
| 5661 | 1111.608 | 30.47 | 7.52 | 4.06 | 8.29 | 5.98 | 7.90 | 5.15 | 1.85 | 1.39 | 1.53 |
| 6367 | 1155.575 | 29.05 | 6.57 | 4.10 | 7.14 | 3.41 | 8.39 | 4.48 | 1.60 | 2.09 | 1.87 |
| 6546 | 1167.54 | 47.90 | 4.49 | 5.59 | 4.63 | 3.92 | 5.08 | 4.30 | 0.80 | 1.18 | 1.18 |
| 7006 | 1196.367 | 47.02 | 12.07 | 12.79 | 12.42 | 13.09 | 12.91 | 13.48 | 0.94 | 0.95 | 0.96 |
| 7982 | 1255.65 | 26.25 | 6.73 | 4.55 | 7.88 | 3.90 | 6.75 | 5.44 | 1.48 | 2.02 | 1.24 |
| 8591 | 1295.622 | 29.62 | 8.05 | 3.67 | 8.18 | 3.00 | 9.73 | 4.23 | 2.19 | 2.73 | 2.30 |
| 9325 | 1341.649 | 38.48 | 6.65 | 5.63 | 6.62 | 5.30 | 7.52 | 5.21 | 1.18 | 1.25 | 1.44 |
| 10270 | 1407.682 | 48.28 | 7.91 | 9.23 | 8.50 | 9.14 | 8.91 | 6.45 | 0.86 | 0.93 | 1.38 |
| 10772 | 1444.684 | 40.04 | 4.60 | 5.84 | 4.28 | 3.39 | 7.37 | 4.59 | 0.79 | 1.26 | 1.61 |
| 11141 | 1471.739 | 39.49 | 4.27 | 4.30 | 4.99 | 4.22 | 5.34 | 4.74 | 0.99 | 1.18 | 1.13 |
| 12407 | 1564.769 | 39.41 | 7.19 | 5.89 | 8.34 | 6.64 | 8.90 | 5.66 | 1.22 | 1.26 | 1.57 |
| 12582 | 1579.76 | 26.34 | 8.60 | 7.18 | 10.10 | 8.95 | 9.86 | 6.87 | 1.20 | 1.13 | 1.44 |
| 12628 | 1584.553 | 47.97 | 10.32 | 12.13 | 11.42 | 12.12 | 11.48 | 12.24 | 0.85 | 0.94 | 0.94 |
| 12969 | 1611.795 | 40.17 | 5.59 | 7.54 | 5.92 | 6.83 | 5.78 | 7.19 | 0.74 | 0.87 | 0.80 |
| 13270 | 1635.838 | 33.09 | 6.11 | 4.45 | 6.30 | 3.66 | 7.01 | 4.56 | 1.37 | 1.72 | 1.53 |
| 13543 | 1659.818 | 39.66 | 8.96 | 6.90 | 9.43 | 6.79 | 9.41 | 5.46 | 1.30 | 1.39 | 1.72 |
| 13580 | 1663.806 | 40.59 | 8.70 | 5.54 | 9.39 | 4.19 | 8.92 | 4.80 | 1.57 | 2.24 | 1.86 |
| 13742 | 1679.814 | 40.76 | 7.82 | 6.23 | 8.45 | 5.75 | 7.40 | 5.22 | 1.25 | 1.47 | 1.42 |
| 14788 | 1780.881 | 28.09 | 6.98 | 4.44 | 9.02 | 2.89 | 9.26 | 4.33 | 1.57 | 3.12 | 2.14 |
| 14896 | 1790.854 | 33.26 | 6.62 | 4.42 | 8.29 | 5.42 | 8.28 | 4.66 | 1.50 | 1.53 | 1.78 |
| 15819 | 1885.854 | 28.11 | 9.79 | 4.57 | 10.90 | 3.50 | 11.42 | 4.68 | 2.14 | 3.12 | 2.44 |
| 100383 | 1139.544 | 28.69 | 9.92 | 6.65 | 10.46 | 7.06 | 10.89 | 6.37 | 1.49 | 1.48 | 1.71 |
| 100433 | 1173.604 | 26.22 | 6.30 | 4.47 | 7.69 | 3.67 | 6.06 | 4.87 | 1.41 | 2.10 | 1.24 |
| 100744 | 1417.721 | 26.35 | 5.87 | 4.27 | 7.21 | 4.15 | 6.42 | 4.65 | 1.37 | 1.74 | 1.38 |
| 100773 | 1433.684 | 32.66 | 7.07 | 5.52 | 7.79 | 6.11 | 7.93 | 6.28 | 1.28 | 1.28 | 1.26 |
| 100916 | 1532.692 | 39.51 | 6.33 | 8.34 | 8.62 | 9.19 | 7.00 | 9.06 | 0.76 | 0.94 | 0.77 |
| 100954 | 1554.759 | 26.62 | 6.33 | 4.60 | 7.18 | 3.57 | 6.80 | 4.57 | 1.38 | 2.01 | 1.49 |
| 100964 | 1561.754 | 28.12 | 7.59 | 4.19 | 8.80 | 3.89 | 8.20 | 4.91 | 1.81 | 2.26 | 1.67 |
| 101001 | 1584.787 | 27.29 | 7.10 | 4.52 | 8.12 | 3.52 | 8.32 | 4.83 | 1.57 | 2.31 | 1.72 |
| 101201 | 1713.872 | 28.25 | 7.19 | 4.43 | 8.82 | 3.54 | 9.03 | 4.84 | 1.62 | 2.49 | 1.87 |
| 101224 | 1726.832 | 28.14 | 5.34 | 4.55 | 5.81 | 3.76 | 6.37 | 4.69 | 1.17 | 1.54 | 1.36 |
| 101391 | 1832.94 | 33.34 | 6.33 | 4.54 | 6.28 | 3.54 | 7.78 | 4.94 | 1.39 | 1.77 | 1.57 |
| 101392 | 1833.646 | 48.36 | 5.17 | 6.55 | 4.94 | 6.58 | 6.78 | 4.81 | 0.79 | 0.75 | 1.41 |
| 101695 | 2013.958 | 25.17 | 8.05 | 4.63 | 8.93 | 3.74 | 9.39 | 5.05 | 1.74 | 2.39 | 1.86 |
| 102001 | 2196.056 | 25.43 | 7.54 | 4.18 | 9.66 | 3.76 | 9.26 | 4.61 | 1.80 | 2.57 | 2.01 |
| 102056 | 2239.077 | 26.87 | 8.01 | 4.65 | 9.33 | 3.52 | 8.34 | 5.06 | 1.72 | 2.65 | 1.65 |
| 106356 | 1418.602 | 48.47 | 7.81 | 4.70 | 7.40 | 3.86 | 8.73 | 5.14 | 1.66 | 1.92 | 1.70 |
| 880 | 855.4258 | 35.91 | 9.61 | 10.94 | 9.42 | 10.64 | 8.86 | 11.32 | 0.88 | 0.88 | 0.78 |
| 1512 | 883.4193 | 35.35 | 7.59 | 8.27 | 7.77 | 6.69 | 8.43 | 6.66 | 0.92 | 1.16 | 1.27 |
| 1615 | 888.4502 | 34.67 | 7.05 | 6.27 | 8.22 | 5.97 | 7.04 | 4.86 | 1.13 | 1.38 | 1.45 |
| 2684 | 931.5043 | 27.26 | 7.97 | 7.67 | 7.49 | 9.31 | 7.82 | 8.83 | 1.04 | 0.80 | 0.89 |
| 2981 | 947.4504 | 34.20 | 7.66 | 7.31 | 7.23 | 5.09 | 7.51 | 5.79 | 1.05 | 1.42 | 1.30 |
| 3258 | 960.5006 | 29.69 | 5.54 | 6.03 | 7.14 | 5.88 | 5.93 | 4.99 | 0.92 | 1.21 | 1.19 |
| 3668 | 982.5227 | 36.14 | 10.42 | 10.22 | 10.41 | 9.58 | 10.30 | 8.68 | 1.02 | 1.09 | 1.19 |
| 3784 | 989.4635 | 47.52 | 6.51 | 6.42 | 6.74 | 7.99 | 7.02 | 5.54 | 1.01 | 0.84 | 1.27 |
| 3791 | 989.5253 | 28.87 | 8.40 | 9.05 | 7.80 | 9.24 | 6.99 | 8.98 | 0.93 | 0.84 | 0.78 |
| 3930 | 998.4752 | 34.70 | 10.08 | 9.68 | 9.56 | 8.17 | 9.43 | 6.41 | 1.04 | 1.17 | 1.47 |
| 4042 | 1004.478 | 47.47 | 7.44 | 8.21 | 7.72 | 8.92 | 8.85 | 6.97 | 0.91 | 0.87 | 1.27 |
| 4680 | 1044.505 | 36.33 | 6.11 | 7.63 | 6.18 | 5.59 | 6.53 | 5.53 | 0.80 | 1.11 | 1.18 |
| 4824 | 1055.523 | 36.37 | 9.66 | 9.55 | 9.44 | 8.94 | 9.49 | 8.91 | 1.01 | 1.06 | 1.06 |
| 5014 | 1068.567 | 29.18 | 5.30 | 4.67 | 5.62 | 3.91 | 5.66 | 4.84 | 1.14 | 1.44 | 1.17 |
| 5114 | 1073.515 | 36.41 | 6.52 | 5.76 | 5.98 | 3.96 | 6.25 | 5.09 | 1.13 | 1.51 | 1.23 |
| 5789 | 1119.531 | 48.01 | 4.82 | 5.38 | 4.67 | 5.91 | 6.64 | 5.91 | 0.90 | 0.79 | 1.12 |
| 6058 | 1134.515 | 47.95 | 6.16 | 4.69 | 4.99 | 5.56 | 7.18 | 5.16 | 1.31 | 0.90 | 1.39 |
| 6252 | 1148.544 | 48.13 | 4.93 | 4.99 | 4.94 | 5.90 | 6.36 | 5.00 | 0.99 | 0.84 | 1.27 |
| 6545 | 1167.564 | 29.66 | 8.30 | 5.88 | 5.88 | 7.50 | 5.67 | 9.18 | 1.41 | 0.78 | 0.62 |
| 7181 | 1206.626 | 25.61 | 6.64 | 5.38 | 6.25 | 7.93 | 7.04 | 5.14 | 1.23 | 0.79 | 1.37 |
| 7302 | 1214.566 | 37.03 | 11.12 | 11.34 | 11.69 | 11.14 | 11.60 | 12.04 | 0.98 | 1.05 | 0.96 |
| 7534 | 1227.606 | 37.47 | 10.57 | 10.37 | 10.26 | 9.45 | 10.65 | 9.95 | 1.02 | 1.09 | 1.07 |
| 8404 | 1283.405 | 47.36 | 6.44 | 6.77 | 7.21 | 3.99 | 8.16 | 6.39 | 0.95 | 1.81 | 1.28 |
| 8590 | 1295.61 | 37.80 | 5.85 | 5.95 | 6.53 | 4.29 | 7.54 | 5.62 | 0.98 | 1.52 | 1.34 |
| 8661 | 1300.715 | 39.78 | 5.99 | 4.46 | 7.22 | 3.86 | 8.35 | 4.88 | 1.34 | 1.87 | 1.71 |
| 9006 | 1321.704 | 32.99 | 6.65 | 6.20 | 6.63 | 5.18 | 7.11 | 5.92 | 1.07 | 1.28 | 1.20 |
| 9090 | 1324.681 | 29.94 | 7.57 | 7.03 | 9.26 | 7.61 | 9.05 | 6.34 | 1.08 | 1.22 | 1.43 |
| 9131 | 1326.631 | 39.17 | 8.23 | 9.06 | 8.20 | 5.14 | 7.11 | 5.30 | 0.91 | 1.60 | 1.34 |
| 9601 | 1358.66 | 32.35 | 7.02 | 7.36 | 5.34 | 6.53 | 6.96 | 5.43 | 0.95 | 0.82 | 1.28 |
| 9863 | 1378.668 | 39.11 | 10.44 | 10.55 | 10.86 | 10.08 | 10.22 | 9.15 | 0.99 | 1.08 | 1.12 |
| 9871 | 1378.731 | 31.31 | 6.94 | 6.50 | 8.40 | 7.60 | 8.35 | 6.65 | 1.07 | 1.11 | 1.26 |
| 10013 | 1388.717 | 39.15 | 9.66 | 9.21 | 10.47 | 9.54 | 9.82 | 8.04 | 1.05 | 1.10 | 1.22 |
| 10393 | 1415.706 | 39.41 | 5.71 | 5.70 | 5.80 | 6.85 | 6.18 | 5.72 | 1.00 | 0.85 | 1.08 |
| 10625 | 1434.691 | 48.95 | 4.71 | 7.65 | 6.15 | 8.04 | 7.28 | 7.07 | 0.62 | 0.76 | 1.03 |
| 11179 | 1474.77 | 26.54 | 4.98 | 4.51 | 5.79 | 4.18 | 5.51 | 4.64 | 1.10 | 1.38 | 1.19 |
| 11487 | 1497.745 | 31.62 | 4.91 | 4.49 | 5.23 | 3.61 | 5.03 | 4.42 | 1.09 | 1.45 | 1.14 |
| 11667 | 1511.678 | 39.29 | 5.95 | 6.83 | 6.13 | 5.35 | 6.38 | 5.45 | 0.87 | 1.14 | 1.17 |
| 11737 | 1515.825 | 32.86 | 4.71 | 6.21 | 6.72 | 5.36 | 4.86 | 5.78 | 0.76 | 1.25 | 0.84 |
| 12757 | 1594.92 | 33.72 | 6.04 | 5.80 | 4.70 | 6.45 | 5.86 | 8.83 | 1.04 | 0.73 | 0.66 |
| 12958 | 1610.734 | 38.89 | 6.83 | 6.16 | 8.06 | 4.90 | 7.45 | 4.53 | 1.11 | 1.65 | 1.65 |
| 13079 | 1620.735 | 32.13 | 5.06 | 4.48 | 5.99 | 3.96 | 6.87 | 4.57 | 1.13 | 1.51 | 1.50 |
| 13290 | 1636.792 | 40.73 | 10.05 | 10.03 | 10.51 | 9.77 | 9.67 | 8.59 | 1.00 | 1.08 | 1.13 |
| 13458 | 1650.839 | 33.29 | 5.19 | 4.89 | 6.68 | 4.79 | 5.98 | 4.59 | 1.06 | 1.39 | 1.30 |
| 13670 | 1671.9 | 28.18 | 7.11 | 6.31 | 8.28 | 6.70 | 6.81 | 4.66 | 1.13 | 1.24 | 1.46 |
| 13740 | 1679.77 | 32.30 | 9.18 | 9.20 | 9.07 | 8.01 | 9.78 | 8.75 | 1.00 | 1.13 | 1.12 |
| 13819 | 1687.84 | 41.42 | 5.26 | 4.85 | 5.32 | 4.24 | 5.58 | 4.59 | 1.08 | 1.25 | 1.22 |
| 14020 | 1706.866 | 41.37 | 7.12 | 8.04 | 8.11 | 7.12 | 7.37 | 5.42 | 0.89 | 1.14 | 1.36 |
| 14404 | 1743.872 | 33.58 | 6.88 | 6.17 | 7.60 | 6.29 | 6.51 | 5.11 | 1.11 | 1.21 | 1.27 |
| 15228 | 1825.811 | 40.93 | 6.65 | 7.42 | 7.23 | 6.53 | 6.96 | 5.55 | 0.90 | 1.11 | 1.25 |
| 16047 | 1905.941 | 34.11 | 8.35 | 7.80 | 9.16 | 7.25 | 6.44 | 5.27 | 1.07 | 1.26 | 1.22 |
| 16054 | 1906.927 | 42.64 | 8.95 | 9.25 | 8.80 | 7.90 | 8.12 | 5.89 | 0.97 | 1.11 | 1.38 |
| 16851 | 1992.943 | 42.36 | 8.77 | 9.29 | 9.21 | 8.36 | 8.20 | 7.30 | 0.94 | 1.10 | 1.12 |
| 16914 | 2000.106 | 35.84 | 8.97 | 9.41 | 7.33 | 9.83 | 7.61 | 11.52 | 0.95 | 0.75 | 0.66 |
| 17594 | 2072.013 | 40.74 | 6.32 | 6.90 | 7.02 | 5.83 | 6.98 | 5.04 | 0.92 | 1.20 | 1.38 |
| 17878 | 2099.082 | 42.32 | 6.14 | 7.02 | 6.11 | 4.23 | 5.76 | 5.15 | 0.88 | 1.45 | 1.12 |
| 20566 | 2420.078 | 44.61 | 7.04 | 7.68 | 7.16 | 5.77 | 6.39 | 4.77 | 0.92 | 1.24 | 1.34 |
| 21602 | 2566.11 | 44.92 | 5.86 | 6.61 | 6.42 | 5.13 | 5.45 | 4.77 | 0.89 | 1.25 | 1.14 |
| 29610 | 3981.973 | 38.00 | 5.17 | 4.45 | 7.19 | 4.64 | 6.16 | 4.40 | 1.16 | 1.55 | 1.40 |
| 33289 | 5452.868 | 24.75 | 11.77 | 11.77 | 10.45 | 12.70 | 12.27 | 8.41 | 1.00 | 0.82 | 1.46 |
| 100000 | 800.3959 | 33.34 | 8.16 | 7.23 | 7.97 | 6.08 | 8.15 | 6.32 | 1.13 | 1.31 | 1.29 |
| 100026 | 828.4175 | 33.50 | 6.76 | 7.59 | 7.67 | 6.31 | 7.10 | 5.33 | 0.89 | 1.22 | 1.33 |
| 100111 | 902.4517 | 29.35 | 6.53 | 5.56 | 5.89 | 4.96 | 5.69 | 4.71 | 1.18 | 1.19 | 1.21 |
| 100343 | 1095.353 | 47.05 | 7.70 | 5.81 | 5.62 | 8.58 | 7.54 | 5.58 | 1.32 | 0.66 | 1.35 |
| 100542 | 1253.576 | 37.28 | 6.04 | 5.74 | 6.71 | 3.38 | 7.61 | 5.45 | 1.05 | 1.98 | 1.40 |
| 100572 | 1274.494 | 47.99 | 5.81 | 5.64 | 7.80 | 5.10 | 7.87 | 5.09 | 1.03 | 1.53 | 1.55 |
| 100806 | 1456.767 | 27.87 | 4.88 | 4.34 | 6.49 | 3.77 | 6.23 | 4.55 | 1.12 | 1.72 | 1.37 |
| 100913 | 1529.64 | 29.08 | 5.58 | 5.96 | 7.43 | 9.05 | 7.38 | 8.35 | 0.94 | 0.82 | 0.88 |
| 101148 | 1675.841 | 41.45 | 5.90 | 5.66 | 6.60 | 3.80 | 6.44 | 4.69 | 1.04 | 1.74 | 1.37 |
| 101272 | 1753.899 | 50.19 | 5.49 | 5.94 | 5.60 | 3.57 | 7.09 | 4.92 | 0.92 | 1.57 | 1.44 |
| 101403 | 1837.903 | 28.25 | 5.91 | 4.33 | 8.18 | 3.73 | 8.58 | 4.47 | 1.37 | 2.19 | 1.92 |
| 101420 | 1849.971 | 29.18 | 5.92 | 4.96 | 7.31 | 5.51 | 5.86 | 4.90 | 1.19 | 1.33 | 1.20 |
| 101782 | 2060.054 | 42.88 | 5.29 | 6.96 | 6.97 | 4.05 | 6.43 | 4.22 | 0.76 | 1.72 | 1.52 |
| 101946 | 2165.127 | 30.74 | 5.07 | 4.46 | 5.74 | 3.55 | 5.90 | 5.22 | 1.14 | 1.62 | 1.13 |
| 102676 | 3411.458 | 28.83 | 5.13 | 5.84 | 5.77 | 5.83 | 5.64 | 4.81 | 0.88 | 0.99 | 1.17 |
| 102804 | 4113.821 | 47.13 | 4.65 | 4.25 | 6.77 | 3.70 | 4.98 | 4.50 | 1.09 | 1.83 | 1.11 |
| 102868 | 4816.223 | 32.08 | 5.01 | 4.66 | 5.19 | 3.56 | 5.06 | 4.41 | 1.08 | 1.46 | 1.15 |
| 107005 | 1694.835 | 33.02 | 5.52 | 4.25 | 6.85 | 4.44 | 6.47 | 4.44 | 1.30 | 1.54 | 1.46 |

# Table S2: Sequenced peptides list

| **PeptideID** | **Mass [Da]** | **Sequence** | **Protein name** | **Theoretical Mass** | **Start AA** | **Stop AA** | **Rat.Protein Accessions** |
| --- | --- | --- | --- | --- | --- | --- | --- |
| 12757 | 1594.92 | IDQTRVLNLGPITR | Uromodulin | 1594.91549 | 596 | 609 | P27590 |
| 16914 | 2000.106 | SGNFIDQTRVLNLGPITR | Uromodulin | 2000.080323 | 592 | 609 | P27590 |
| 9131 | 1326.631 | TVDETYVPKEF | Serum albumin | 1326.634348 | 516 | 526 | [P02770](http://www.uniprot.org/uniprot/P02770) |
| 11179 | 1474.77 | SVIHEDVYEEKK | RCG32337, isoform CRA_a | 1474.730374 | 47 | 58 | D3ZJA4 |
| 14788 | 1780.881 | DKTEKELLDSYIDGR | Prothrombin | 1780.884309 | 345 | 359 | P18292 |
| 3668 | 982.5227 | VPSYPGPpGP | Protein Col19a1 | 982.569898 | 76 | 84 | D3ZCQ0 |
| 8590 | 1295.61 | DPVESKIYFAQ | Pro-epidermal growth factor | 1295.639768 | 522 | 532 | [P07522](http://www.uniprot.org/uniprot/P07522) |
| 11667 | 1511.678 | AGPpGPpGpPGSIGHpG | procollagen, type IX, alpha 3 (predicted), isoform CRA_a | 1511.700471 | 555 | 571 | D3ZX71 |
| 12958 | 1610.734 | LAQLmANEWPHSQA | NACHT, leucine rich repeat and PYD containing 5 | 1610.751105 | 69 | 82 | D3ZDM5 |
| 9006 | 1321.704 | DGILGRDTLPHE | Contrapsin-like protease inhibitor 1 | 1321.662629 | 21 | 32 | [P05545](http://www.uniprot.org/uniprot/P05545) |
| 11141 | 1471.739 | ALYQAEAFVADFK | Contrapsin-like protease inhibitor 1 | 1471.734731 | 155 | 167 | [P05545](http://www.uniprot.org/uniprot/P05545) |
| 11487 | 1497.745 | GPPGpPGDPGKPGAPGK | Collagen alpha-1(IX) chain | 1497.757592 | 68 | 84 | F1LQ93 |
| 13742 | 1679.814 | GMpGSpGGPGNDGKPGPpG | Collagen alpha-1(III) chain | 1679.720948 | 536 | 554 | P13941 |
| 14896 | 1790.854 | GESGRpGPpGPSGPRGQpG | Collagen alpha-1(III) chain | 1790.829588 | 557 | 575 | P13941 |
| 16851 | 1992.943 | QGIpGTSGPpGENGKpGEpGP | Collagen alpha-1(III) chain | 1992.902478 | 640 | 660 | P13941 |
| 4706 | 1046.479 | GppGPpGPpGPG | Collagen alpha-1(II) chain | 1046.466889 | 1139 | 1150 | P05539 |
| 9863 | 1378.668 | ApGEDGRpGPpGPQ | Collagen alpha-1(II) chain | 1378.611322 | 512 | 525 | P05539 |
| 10625 | 1434.691 | GPpGPpGPpGPPSGGY | Collagen alpha-1(I) chain | 1434.641559 | 1170 | 1185 | P02454 |
| 3784 | 989.4635 | GppGPpGPpGP | Collagen alpha-1(I) chain | 989.445425 | 131 | 141 | P02454 |
| 4042 | 1004.478 | GPpGPpGPPSGG | Collagen alpha-1(I) chain | 1004.456324 | 1173 | 1184 | P02454 |
| 4119 | 1009.498 | GRVGPpGPSGN | Collagen alpha-1(I) chain | 1009.494075 | 870 | 880 | P02454 |
| 6546 | 1167.54 | GPpGPpGPPSGGY | Collagen alpha-1(I) chain | 1167.519653 | 1173 | 1185 | P02454 |
| 4680 | 1044.505 | ApGFpGARGPS | Collagen alpha-1(I) chain | 1044.498815 | 397 | 407 | P02454 |
| 5254 | 1083.533 | GVVGLpGQRGE | Collagen alpha-1(I) chain | 1083.483267 | 828 | 839 | P02454 |
| 5789 | 1119.531 | GPpGPTGPTGPpG | Collagen alpha-1(I) chain | 1119.519653 | 321 | 333 | P02454 |
| 6252 | 1148.544 | GLpGPpGApGPQG | Collagen alpha-1(I) chain | 1148.546202 | 177 | 189 | P02454 |
| 9090 | 1324.681 | GLpGpKGDRGDAGP | Collagen alpha-1(I) chain | 1324.637142 | 726 | 739 | P02454 |
| 10013 | 1388.717 | RpGEVGPpGPpGPAG | Collagen alpha-1(I) chain | 1388.668442 | 907 | 921 | P02454 |
| 11737 | 1515.825 | GPpGPpGPVGKEGGKGP | Collagen alpha-1(I) chain | 1515.768157 | 882 | 898 | P02454 |
| 13079 | 1620.735 | DGVAGPKGPAGERGSpGP | Collagen alpha-1(I) chain | 1620.785598 | 488 | 505 | P02454 |
| 13290 | 1636.792 | GSpGSpGPDGKTGPpGPAG | Collagen alpha-1(I) chain | 1636.732893 | 531 | 549 | P02454 |
| 13670 | 1671.9 | PpGPpGPVGKEGGKGPRG | Collagen alpha-1(I) chain | 1671.869268 | 882 | 899 | P02454 |
| 14020 | 1706.866 | TGPIGPpGPAGApGDKGET | Collagen alpha-1(I) chain | 1706.811144 | 755 | 773 | P02455 |
| 13543 | 1659.818 | GAPGAKGNVGppGEPGPpG | Alpha 4 type V collagen | 1659.785263 | 620 | 638 | P68136 |
| 4824 | 1055.523 | NELRVAPEE | Actin, alpha skeletal muscle | 1055.524738 | 94 | 102 | P68136 |

Table S3: Proteasix prediction

# Supplementary figure S1: Nifedipine significantly inhibits blood pressure elevation in the SHRSP. Systolic (SBP) (A) and diastolic (DBP) (B) blood pressure was monitored in untreated WKY, untreated SHRSP and nifedipine treated SHRSP (n=6) using radiotelemetry before day 0 and during pregnancy (gestational day 0 – day 21). SHRSP had significantly increased blood pressure compared to WKY (* p<0.05). Nifedipine treatment significantly reduced SHRSP blood pressure (## p<0.01 vs. SHRSP). Data analysed by comparing area under the curve values using one way ANOVA and Tukey’s post-hoc test.
